# Age Differences in Older Adults’ Usage of Selection, Optimization, and Compensation: Findings from the SONIC Study

**DOI:** 10.1093/geroni/igaf122.3008

**Published:** 2025-12-31

**Authors:** Momoho Kakuta, Takeshi Nakagawa, Yukie Masui, Kei Kamide, Kazunori Ikebe, Takumi Hirata, Yasumichi Arai, Yasuyuki Gondo

**Affiliations:** The University of Osaka, Suita, Osaka, Japan; The University of Osaka, Osaka, Osaka, Japan; Tokyo Metropolitan Institute of Gerontology, Itabashi City, Tokyo, Japan; Osaka University, Osaka, Osaka, Japan; Osaka University, Osaka, Osaka, Japan; Tokyo Metropolitan Institute of Gerontology, Itabashi, Tokyo, Japan; Keio University Graduate School of Health Management, Fujisawa, Kanagawa, Japan; The University of Osaka, Suita, Osaka, Japan

## Abstract

Selection, Optimization, and Compensation (SOC) were life-management strategies to maintain well-being against function declines. The number of SOC strategies decreased from middle to old age due to fewer resources. However, little is known about the change in SOC strategy from young-old to very old despite a highly significant resource decrease. This study aimed to clarify the age differences in SOC strategy. Data collected from community-dwelling Japanese people participated in the longitudinal cohort study of Septuagenarians, Octogenarians, and Nonagenarians Investigation with Centenarians (SONIC). The samples were divided into 70s (n = 983, 48.2% male, range:68-72), 80s (n = 957, 47.1% male, range:78-82), and 90s (n = 212, 47.2% male, range:88-92). We conducted multiple group factor analysis using polychoric correlation, ANOVA, and correlation analysis between SOC subfactors, subjective well-being, personality traits, and cognitive function. Model fit indices were adequate to compare scores with three generation cohorts (CFI=.960, TLI=.945, RMSEA=.041, SRMR=.078). Only the use of elective selection was significantly higher with aging, but other subfactors were higher from the 70s to 80s and were lower from the 80s to 90s. The only elective selection was negatively correlated (r =-.09 -.01), and other subfactors were positively correlated (r =.02 .30) with well-being in the 70s and 80s, but the correlation coefficients were weaker in the 90s, respectively (r =-.09 .13). We emphasized that the use and the impact of SOC was maximized until the 80s and changed in the 90s. The key factor of well-being in the 90s was not only the SOC strategy.

